# Come from away: Best practices in mini-sabbaticals for the development of young investigators: a White Paper by the SEQUIN (mini-Sabbatical Evaluation and QUality ImprovemeNt) Group

**DOI:** 10.1017/cts.2019.369

**Published:** 2019-06-20

**Authors:** Michael H. Pillinger, Stephenie C. Lemon, Martin S. Zand, P. Jeffrey Foster, Jeanne S. Merchant, Robert Kimberly, Jeroan Allison, Bruce N. Cronstein, Claudia Galeano, Jeanne Holden-Wiltse, Melissa Trayhan, Robert J. White, Amanda Davin, Kenneth G. Saag

**Affiliations:** 1 The Clinical and Translational Science Institute, New York University School of Medicine, New York, NY, USA; 2 The Center for Clinical and Translational Science, University of Massachusetts School of Medicine, Worcester, MA, USA; 3 The Center for Leading Innovation and Collaboration and Clinical and Translational Science Institute, University of Rochester School of Medicine, Rochester, NY, USA; 4 The Center for Clinical and Translational Science, University of Alabama School of Medicine, Birmingham, AL, USA

**Keywords:** Mini-sabbatical, sabbatical, education, training, skills development, TL1, KL2

## Abstract

Mini-sabbaticals are formal short-term training and educational experiences away from an investigator’s home research unit. These may include rotations with other research units and externships at government research or regulatory agencies, industry and non-profit programs, and training and/or intensive educational programs. The National Institutes of Health have been encouraging training institutions to consider offering mini-sabbaticals, but given the newness of the concept, limited data are available to guide the implementation of mini-sabbatical programs. In this paper, we review the history of sabbaticals and mini-sabbaticals, report the results of surveys we performed to ascertain the use of mini-sabbaticals at Clinical and Translational Science Award hubs, and consider best practice recommendations for institutions seeking to establish formal mini-sabbatical programs.

In 2013, an Institute of Medicine report encouraged Clinical and Translational Science Award (CTSA) programs to develop and implement innovative curricula focusing on team-based experiential education and training to enhance the ability of investigators to work in multidisciplinary research groups [1]. In response, the National Center for the Advancement of Translational Science (NCATS) and other federal training programs called upon institutions applying for training awards to consider offering non-traditional training experiences, which they termed “mini-sabbaticals” [2]. Mini-sabbaticals, including short-term rotations, intensive courses, and externships, are intended to enrich career development through brief experiences complementary to those offered at the investigator’s home institution. Venues may include academic institutions, industry, regulatory agencies, non-profit, and other research stakeholder organizations. The goal of the mini-sabbatical experience is to acquire added competencies and collaborations in specific areas of translational research in a manner tailored to meet individual training needs, and potentially to provide opportunities for future collaboration. Mini-sabbaticals represent a relatively new concept in research training that has been promulgated by multiple federally funded training programs, including the Ks and Ts supported by the Agency for Healthcare Research and Quality (AHRQ) [3–7]. For CTSA institutions, with their commitment to K and T training and to career development generally, the notion of a mini-sabbatical may be particularly appealing.

While mini-sabbaticals hold potential for promoting team and multidisciplinary science, enhancing cross-CTSA collaboration and leveraging the collective expertise of the national CTSA network, their utility in practice has yet to be examined. Questions to be answered about mini-sabbaticals include: (1) What constitutes optimal content and format? (2) What types of training gaps are best addressed by these experiences? (3) How often, where, and when should developing translational investigators complete one? and (4) What opportunities already exist nationally and should be developed through the CTSA, and how can investigators identify and access them?

To begin to address these and other questions, we established the mini-Sabbatical Evaluation and QUality ImprovemeNt (SEQUIN) group. Partner CTSA hubs include the University of Alabama School of Medicine at Birmingham (UAB), the University of Massachusetts School of Medicine (U Mass), New York University–New York City Health and Hospitals (NYU-HH), and the Center for Leading Innovation and Collaboration (CLIC) at the University of Rochester School of Medicine. The goals of SEQUIN have been to describe current mini-sabbatical practices within the CTSA network, to define best practices in mini-sabbatical offerings and mechanisms based on the available literature and formative input from CTSA training leaders and trainees who have experienced mini-sabbaticals, and to develop a system for disseminating CTSA-based mini-sabbatical opportunities nationally. Here, we describe our efforts to date, recommend best practices, and report on our progress in promoting nationwide discussion and participation.

## Sabbaticals: Historical Perspective

Although other early cultures (e.g., Mesopotamia) may have endorsed occasional holidays, the notion of a regular Sabbath appears to have originated in the Hebrew Bible both as a divine rest from the process of creation [8] and as a recurring human obligation [9]. Thus, the early “sabbatical” was a period of rest and recovery. Later tradition considered the Sabbath as a day for study, combining the obligation to step away from the routine work with the privilege of protected thought and learning. Tradition further enshrined the notion of a sabbatical year, in which fields were allowed to lie fallow every seventh year, and debtors and servants were granted amnesty [10]. Thus, the sabbatical came to imply a turning point, in which new and better directions are taken. In this context, some scholars view the concept of Sabbath as emphasizing both “the idea of personal freedom” and the individual’s “higher mission as a member of the human race” [11].

In American education, the prevailing concept of academic sabbatical began at Harvard, followed quickly by Cornell and Wellesley, and at least seven additional institutions by the end of the 19^th^ century [12]. Based on perceived benefit, the number of institutions offering sabbaticals continued to grow, and by the early 20^th^ century, more than 100 institutions offered sabbaticals. The concept of academic sabbatical leave traditionally included four elements: (1) purpose (i.e., leave with a definite goal); (2) compensation (to make such leave possible); (3) prior service in the institution (i.e., sabbaticals as both earned and necessary, after potentially exhausting service, thus relating specifically to established faculty), and (4) an expectation by the institution that the self-improvement the sabbatee (i.e., sabbatical participant) seeks will benefit the institution as well, often including required post-sabbatical service. Thus, enshrined in the concept of the academic sabbatical is the notion that faculty must be given opportunities to grow, in order that their institutions may grow as well.

## Benefits and Limitations of Traditional Sabbaticals

Data that document sabbatical benefits are limited. In one study of established physician faculty, taking a sabbatical was associated with clinical but not academic promotions [13]. However, a more extensive review of 52 sabbaticals at the same institution found that sabbatical leave was associated with 89 peer-reviewed publications, 16 professional (e.g., clinical role) promotions, and 20 academic (e.g., faculty title) promotions; 94% of sabbatees reported that they applied what they learned upon return to their institutions [14]. In another retrospective study of a leave program for established physician faculty, sabbaticals were associated with enhanced professional development; post-sabbatical, many sabbatees expanded their institutional commitments and assumed leadership roles [15]. Well-being and overall personal “resources” (e.g., skills, expertise, resilience) may increase for sabbatees compared with others [16]. Sabbatees may be more likely to reflect on their careers, develop new research interests, and provide enhanced benefit to their home institution [17].

Traditional extended sabbaticals are not without drawbacks. For the potential sabbatee, an extended period away may bring concerns about losing momentum in ongoing research and academic activities, and social strains on themselves and their family. For the institution, the extended departure of a valued faculty member can have implications on the administrative and educational duties the sabbatee normally manages, and on the faculty members who have to assume duties for their colleague [18]. Additionally, the cost of an extended sabbatical is a limiting factor, with most sabbatical programs, therefore, geared toward senior and/or tenured faculty, depriving more junior faculty of the opportunity to benefit. Finally, data supporting the academic benefit of sabbaticals to the sponsoring institutions are also limited. Particularly for research faculty and their institutions, the outcomes in terms of institutional prestige and resources remain practically undocumented.

## Mini-Sabbaticals: A Pragmatic Alternative?

**Fig. 1.**
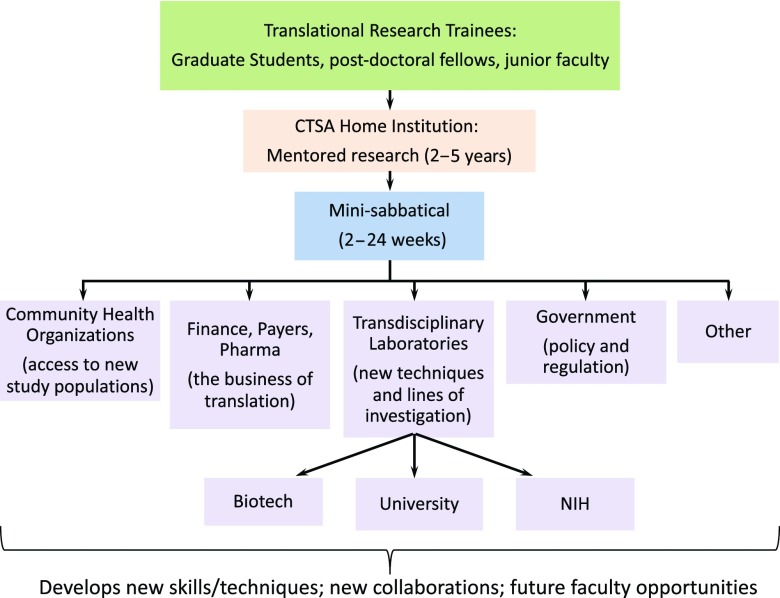
Potential role and utility of mini-sabbaticals for translational research trainees. Trainees committed to an extended but finite period of training at their home institution may benefit from a brief training period away. Carefully targeted experiences in laboratories where they can learn new skills and disciplines, in communities different from the one around their institution, and in the business of translation or in the realms of policy and regulation may all improve the skills and capacities of the junior investigator, provide opportunities for collaboration, and help identify future career pathways. CTSA = Clinical and Translational Science Award. NIH = National Institutes of Health.

In contrast to traditional sabbaticals, mini-sabbaticals may be more practical and potentially more useful, particularly for junior faculty and trainees. While brief trainings away may limit the potential for truly life-changing experiences, they nonetheless offer the possibility of developing specific skills and interests, learning about approaches outside of one’s existing experience, and obtaining insights into future directions for individual research trajectories (Fig. 1). Moreover, they do so with little adverse impact on the quotidian responsibilities of the participant, the workflow needs of the parent institution, or the economic bottom line. Once again, however, the literature is limited and is focused almost entirely on established investigators. Several reflective memoirs from senior faculty attest to the potential value of a mini-sabbatical. Mulder reported on a 4-month mini-sabbatical studying thoracoscopic practices in Europe, from which he came away with the ability to develop new surgical programs and a commitment to international collaboration [19]. In a different vein, Gibb proffered the value of a 2-week mini-sabbatical in which a principal investigator (PI) spends time with another investigator’s laboratory to develop perspectives on “meta-research,” that is, the skills and structures (including administrative and technological expertise) needed to support and organize a lab or research group [20].

For junior workforce trainees, the utility, value, and practicality of a full sabbatical are even more problematic than for established faculty. Being on time-limited contracts, any extended absence from their institutions would be counterproductive, and the expense not likely to be one that institutions would readily bear. Nonetheless, there are good arguments for at least some trainees taking some time away from their base of training. In the increasingly interdisciplinary environment that is research, PI mentors may lack specific skills or methodological expertise that their trainees need to move projects forward. And in the increasingly collaborative environment that is research, trainees need opportunities to meet, and network with, potential future mentors and collaborators in a deeper experience than can be achieved at national conferences. Thus, mini-sabbaticals offer trainees the possibility of acquiring skills, establishing collaborations, and setting the stage for future career opportunities. But what makes a mini-sabbatical successful? And are there best practices that could help ensure that programs seeking to develop or offer mini-sabbaticals do so to the best advantage of themselves and their trainees?

## Mini-Sabbaticals: Defining the State of the Environment

To understand the current status of mini-sabbaticals within the CTSA community, we surveyed CTSA leadership and users of mini-sabbatical programs. We defined a mini-sabbatical as any full-time program away from the trainee’s primary research or training unit, and generally but not exclusively away from the trainee’s institution. We included rotations with other research units or universities, externships of virtually any sort (e.g., government research or regulatory agencies), and industry and non-profit programs and experiences. We further included formal enrichment experiences, such as training and/or intensive educational programs, if they met the other requirements. We excluded scientific meetings and similar experiences that trainees were already likely to attend in the normal course of their training, and defined the temporal maximum of a mini-sabbatical as no more than 6 months.

**Fig. 2.**
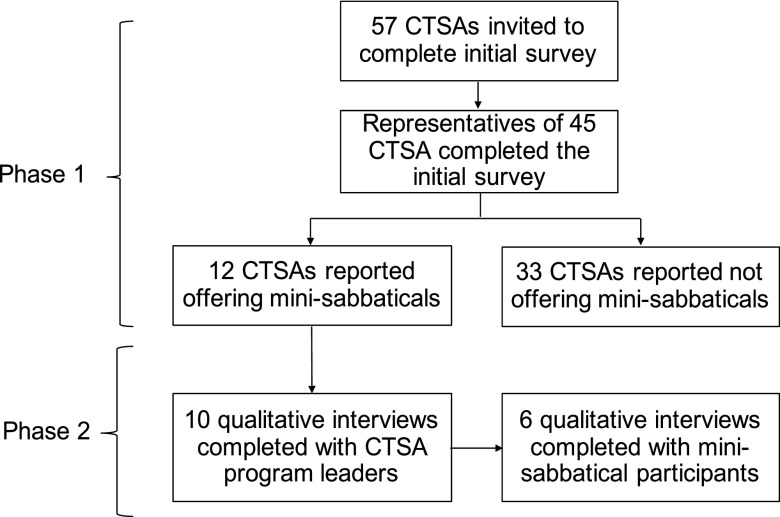
Study overview (CTSA = Clinical and Translational Science Award).

We employed a five-stage approach. First, we internally surveyed our participating SEQUIN partners to identify themes and develop a broadly relevant approach. Second, we conducted a web-based survey of all CTSA institutions (Fig. 2). Email invitations (and, if necessary, up to three reminder emails) were sent by NCATS officials to listservs of the CTSA PIs/directors, operations directors, and educational program leaders. Email invitations included a link to a REDCap survey. Individuals who responded that mini-sabbaticals were available through their CTSA were asked to provide contact information for one or more individuals with knowledge of their program to be contacted for an interview.

Third, we conducted semi-structured key informant interviews with CTSA representatives at the institutions identified in the web-based survey. These individuals were contacted via an email that described how their information was obtained and the purpose of the interview. Again, up to three email reminders were sent. Telephone interviews were performed with willing participants, guided by semi-structured scripts, including 12 core questions ascertaining the nature of their mini-sabbatical programs, administrative procedures used to implement the program (including recruitment, financing, reporting, and evaluation), and barriers and facilitators to offering mini-sabbaticals.

Fourth, based on referrals from program directors, we performed semi-structured key informant interviews with trainees who had participated in mini-sabbatical programs. Trainees were invited either by an email from their CTSA program directors or the SEQUIN investigator, depending upon program director preference. A 12-item guided interview elicited responses to questions ascertaining the nature (i.e., topic, host institution, format, length) of their mini-sabbatical experience, their experiences with administrative components of the program, outcomes of their experience, and positive and negative aspects overall.

Finally, a preliminary set of data was presented to CTSA-affiliated training and education directors at the 2018 Association for Clinical and Translational Science meeting in Washington, DC, with feedback and discussion provided.

*Internal review of SEQUIN collaborating institutions* – A comprehensive review of the SEQUIN institutions (UAB, U Mass, and NYU-HH) identified 20 programs meeting the definition of a mini-sabbatical. In most cases, these represented formalized offerings of brief internship experiences. For the UAB hub, which involves multiple partners distributed throughout the Deep South, such internships often represented “away electives” within the UAB CTSA itself. A single offering constituted a formal, certificate-offering educational program. We identified five additional mini-sabbaticals at academic institutions away from the CTSA hubs, four opportunities in industry, one at a government agency, two in health care systems, and two in non-profit and community partners. All represented relationships established between the hub and the other entity. In many cases, mini-sabbaticals were formalized versions of previously informal internships offered by programs or research units that had an interest in accepting visiting trainees.

In a web-based survey, participants in these programs (29% postdoctoral fellows and 59% assistant professors) had generally positive opinions of their experiences, reporting that the expectations they had for the program were usually achieved, that they would recommend the experience to a colleague, and that the mini-sabbatical contributed to their career development. Most participants also reported that the experience had led to ongoing mentorships and/or collaborations, as well as increased expertise. A substantial minority reported that the experiences, in and of themselves, had led to publication, presentation, and/or funding. Thus, the experience in these institutions – admittedly ones already committed to the mini-sabbatical mechanism – was generally positive.

*National survey and key informant interviews* – Fifty-seven active CTSA hubs were queried in the national web-based survey. Representatives from 45 (87%) responded, with 12 (21%) reporting formal mini-sabbatical programs and the remaining 33 (79%) not offering such programs (Fig. 2).

We completed telephone interviews with faculty representatives from 10 of the 12 programs reporting formal mini-sabbatical programs. Individuals interviewed included CTSA TL1 and KL2 PIs, leaders of mini-sabbatical programs, and other educational directors within the CTSAs. Inductive thematic analysis was used to identify common themes in response to the semi-structured questions [21].

Several themes emerged. First, two mini-sabbatical administrative models predominated. In the first (offered in 8 of 10 CTSAs interviewed), regional networks (both formal and informal) that had sprung up across CTSA institutions led to opportunities for cross-pollination through mini-sabbaticals. In the second, individual CTSA institutions made arrangements for programs with industry, government, and the like (offered in 3 of 10 CTSAs interviewed). Within these models, two approaches to identifying mini-sabbatical opportunities were observed (Table 1). In the first, trainees identified their own training needs and developed their mini-sabbatical experience with administrative assistance (5 of 10 CTSAs interviewed). In the second, opportunities were developed centrally by administration based on institutional areas of strength (6 of 10 CTSAs interviewed). Respondents from two institutions described having also developed websites listing potential mini-sabbatical offerings.

**Table 1.** Mini-sabbatical models identified in the mini-Sabbatical Evaluation and QUality ImprovemeNt (SEQUIN) project utilizing triangulated data from program director (n = 10) and scholar (n = 6) qualitative interviews

Characteristics of program	Scholar self-identified experiences	Institutional offerings in collaborations with other academic institutions, industry, or other stakeholders
Key components	For KL2 scholars, determined in application or after funding received Funded through scholar’s KL2 budget Typically 2 days to 2 weeks in length	Formal arrangements advertised by institution and/or KL2 program Formal application process Typically 2 days to 2 weeks in length
Positive experiences	Administratively easy to implement Relatively inexpensive Networking opportunities Ability to learn new skills and/or content	Institutional offerings may stimulate scholars to explore previously unconsidered possibilities Networking opportunities Ability to learn new skills and/or content Exposure to non-traditional skills and cultures (e.g., industry offerings)
Challenges	Budgeting may only be available to KL2 scholars Limited success in developing collaborative projects Coordinating scheduling of faculty at hosting institution (burden falls on scholars)	Relatively little uptake from scholars (particularly for arrangements with other academic institutions) Limited success in developing collaborative projects Coordinating scheduling of faculty at hosting institution (burden falls on scholars or administrators, depending upon institutions)

Virtually all programs reported that mini-sabbaticals had been developed around, and for, KL2 programs, largely reflecting the inclusion of mini-sabbaticals as a potential component within the NCATS CTSA RFA in September 2014, accompanied by the possibility of dedicated funds to support scholars’ travel and living expenses. All programs reported that most or all of the mini-sabbaticals they developed were brief – days to weeks. Nonetheless, all programs that offered pre-arranged mini-sabbaticals across CTSAs reported that they were utilized at a low rate, possibly reflecting a mismatch between individual trainee needs and pre-arranged offerings.

Overall, the general consensus appeared to be that mini-sabbaticals were a useful and relatively inexpensive program, with the uniform enthusiasm among institutions that included self-directed programs. However, programs reported limited capacity for large-scale initiatives without larger investments from the home institution. In addition, programs made little attempt to formally evaluate the outcomes of these experiences, given the small number of participants at each institution.

Interviews with trainees who had participated in mini-sabbaticals yielded generally positive impressions. Interviewed trainees – all of whom were KL2 scholars – all reported that applying for mini-sabbaticals had been straightforward, that administrative support had been available, and that opportunities for both skill building and networking were valuable. Participants cited the usefulness of institutional structures in helping them secure their mini-sabbatical opportunities. However, participants also noted limitations. Some (4 of 6) reported that the scheduling of their mini-sabbaticals had been challenging, including the need to “time” their program to minimize interruption of regular work and home-lives; trainees with clinical responsibilities reported the greatest challenges. One trainee suggested that a greater number of opportunities, and a better system of access, would be helpful, asking for “more expansion within the … network so that logistically these things are easier to search out.” Interestingly, most (4 of 6) trainees lamented a lack of financial support to permit them to continue collaborative projects after the mini-sabbatical was over.

*Face-to-face discussion with CTSA educational leadership* – A preliminary version of the data reported above was presented during a meeting of KL2 and TL1 leadership at the 2018 Association for Clinical and Translational Science meeting. In the ensuing discussion, several additional themes emerged. First, program directors confirmed that funding mini-sabbaticals remained a challenge, from the point of view of supporting both formalized programs and, perhaps more important, trainees who wished to go away to, or come from, another institution. Issues of travel, housing, and living expenses were paramount, with grant funds limited to KL2 programs and many institutions reporting inadequate discretionary funds to support non-KL2 trainees. Educators were uncertain whether it was more appropriate for the “sending” or “receiving” institution to provide support to the trainee. Some institutions suggested that if NCATS wished to promote mini-sabbaticals, it would be helpful if some costs could be built into the larger CTSA grant structure (rather than the KL2-specific budgets). Concern was expressed about the time constraints of early career faculty and a worry that mini-sabbatical programs should not become a CTSA “unfunded mandate.”

A second theme – consistent with the two approaches reported by CTSA hubs in the survey – was the question of supply-side versus demand-side approaches to mini-sabbatical development. While the SEQUIN group has largely focused on developing programs that trainees could avail themselves of (supply-side approach), several CTSA leaders from non-SEQUIN institutions opined that it would be better to create mechanisms to support trainees who identify their own mini-sabbatical experiences (demand-side approach), based on their positive experiences with this approach. In this way, the individual can customize the away program to their own needs (e.g., identifying a lab group to spend time with, or an intensive course useful to their education) and then apply for funding, contingent upon a convincing application. A noteworthy example of this approach is offered by the Boston University (BU) Clinical and Translational Science Institute, which offers up to three mini-sabbaticals annually “to support BU’s basic researchers, patient-oriented researchers, and population-based researchers working in all areas of translational research related to the prevention, diagnosis, and management of human disease” [22]. Mini-sabbaticals in the BU program are awarded by competitive application, must take place in the USA, and are supported for up to 3 months and 6000 dollars. Since the funding in such programs is not tied to the trainee’s grant, the cost is entirely the burden of the funding CTSA hub, but the management of the program is limited to evaluating and funding proposals rather than maintaining either databases or the mini-sabbaticals themselves.

## Mini-Sabbatical Best Practices and Opportunities

The experiences of the faculty and mini-sabbatical participants described above raise potential challenges and suggest best practice opportunities for institutions wishing to establish such programs. These may be divided into recommendations for the development of individual mini-sabbatical experiences, the structuring and management of overall programs, and the opportunities for funding and disseminating information regarding opportunities (Table 2).

**Table 2.** Best practices for mini-sabbaticals, based on Clinical and Translational Science Award (CTSA) survey

Topic	Recommendation	Comment
Developing mini-sabbaticals to offer		
Duration	Typically, 4–14 days	
Topic(s)	When developing topics, focus on common skills that can be of interest to many junior investigators	Faculty with specific skills may establish more narrowly focused mini-sabbaticals; these may take time to develop an audience
Goals of experience	Highly focused and clearly stated	Institutions should “play to strengths”
Activities	Directly related to goals	Should include opportunities for networking and developing durable relationships, promoting trans-institutional collaborations
	Directly applicable to translational research	
Supporting trainees wishing to design their own “away” mini-sabbaticals	Institutions should develop internal application processes to help ensure that trainees going “away” will have positive experiences	Consider whether mini-sabbaticals developed by trainees may be suitable for future formal offering, in collaboration with the away institution
Organization and administration	Director and administrator assigned to identify and/or develop mini-sabbaticals and oversee mechanisms to vet applicants for in-house and away programs	Consider outreach to local and regional organizations of relevance to your research mission
	Searchable, web-based clearinghouses of mini-sabbatical offerings will help trainees find opportunities	May add to established Center for Leading Innovation and Collaboration (CLIC) database of mini-sabbatical offerings
	Searchable clearinghouses should include contact information of faculty/institutions offering programs, as well as contact information of willing past participants	Outreach to specific groups of interest (e.g., T and K awardees) may help increase the applicant pool
Application processes	Applicants should provide level of training, their goals for the experience, and qualifications	Copies of grant applications (e.g., T and K applications), curricula vitae, and support letters from principal investigator or department should be requested
	Accepted applicants should sign learning agreement with mini-sabbatical director	
Assessment	Immediate assessment of trainee experience after program	Long-term follow-up: did the trainee develop grants and publications relating to mini-sabbatical training? Extended collaborations?

*Developing individual mini-sabbaticals* – Based on survey data and practical funding issues, we recommend that most mini-sabbaticals take place over a period ranging from 4 to 14 days. The duration of the mini-sabbatical should be clearly stated, along with any flexibility for longer or shorter experiences. Mini-sabbaticals of a longer duration (1–6 months) can be undertaken, but require a strong commitment on the part of both the host and trainee institutions. Longer mini-sabbaticals are likely to experience less demand and may best be considered in the context of custom-designed exchange programs. Given their short durations, the goals and activities of each mini-sabbatical should be highly focused, clearly stated and structured, and appropriate to the scale of the mini-sabbatical. Sites or organizations developing mini-sabbaticals should “play to their strengths,” that is, should offer experiences they are distinctly qualified to offer. Individual sites may offer multiple mini-sabbaticals based on their expertise. The precise format of the mini-sabbatical will depend on the expertise and resources of the site and the goal of the experience, potentially including externships, brief formal training experiences, intensive boot camps, and certificate programs. Overall, and to preserve the goal and identity of the program, opportunities should be directly applicable to translational research.

*Supporting trainee needs for customized mini-sabbaticals* – Based on the fact that many mini-sabbaticals are trainee-generated, we recommend that institutions also develop mechanisms to support trainees wishing to propose their own away experiences and mechanisms for ensuring their appropriateness. An application process in which trainees propose and explain their mini-sabbatical plan may suffice. In this context, mini-sabbaticals developed for individual trainees on the “demand-side” principle may deserve future consideration as “supply-side” mini-sabbaticals, that is, it may be appropriate to offer these same experiences to future candidates through formal clearinghouse and application mechanisms.

*Organizing at the CTSA hub level to support mini-sabbaticals* – CTSA hubs wishing to create a mini-sabbatical program should take an active approach to the creation and structuring of their system. Successful programs will require a director, typically someone already knowledgeable about the training component of the CTSA hub. Particularly in the startup phase, this director, with the assistance of administrative support (again, ideally from within the educational unit), will need to identify potential mini-sabbaticals, perform outreach to the potential leaders, and ensure that mini-sabbaticals can meet the standards alluded to above. Based on the assessment of institutional strengths, the director may identify existing degree programs, training programs, and externships to shape into mini-sabbatical offerings (e.g., developing a short course in an area where a semester-long program is currently offered may leverage readily available faculty expertise and resources). The program director should also consider potential opportunities in the community and invite appropriate local organizations to participate, after confirming that they can support the mini-sabbatical format.

In creating mini-sabbaticals, the program director must acknowledge that trainee needs can be highly particular, and that demand for any one mini-sabbatical may be limited. It may, therefore, be a good idea to place initial emphasis on developing mini-sabbaticals with broad potential appeal (e.g., skills-based rather than topic-based experiences). For each mini-sabbatical established, a written contract should be drawn up between the hub and the mini-sabbatical director or site. Such a document would outline the agreement to host trainees, the type and duration of experience to be offered, and what the performance expectations will be for visiting trainees.

Ideally, visiting trainees should have access not only to the mini-sabbatical itself but also to experiences introducing them to the institution and the institution’s staff. Thus, each mini-sabbatical will also have the potential to facilitate trans-institutional collaboration.

*Application/acceptance processes* – Successful mini-sabbatical programs will establish regular rules and application processes, and participate in other network opportunities. It is strongly recommended that each hub creates a web-based clearinghouse of its mini-sabbatical offerings, so that potentially interested trainees (local and distant) can search for, and identify, mini-sabbaticals that meet their needs. At these points of access, programs should list their rules and application processes, provide contact information for questions, and, where possible, offer a roster of former trainees, with contact information, who are willing to share their experiences. Additionally, CTSA hubs may consider reaching out to specific groups likely to be interested in their offerings (e.g., T and K awardees), for example, through e-mailing to the network of CTSAs.

Applicants from outside institutions should provide information regarding their level of training, their institution, a statement of their goals and objectives, and a confirmation of their qualifications to succeed. We recommend that T and K scholars, and those with equivalent awards, also provide a copy of their grant (e.g., Career Development Award) proposal. In the absence of such a grant, applicants should provide a curricula vitae or biosketch. Additionally, applicants should provide a letter of support from their home division/department and PI, confirming their support of the candidate’s application for a mini-sabbatical, including protected time and funds as needed. Applications should be considered twice – first, by the program administration, who ascertain the general appropriateness of the application; and second, by the director of the mini-sabbatical itself – to ensure that the applicant is qualified and that their needs can be met. If there is any doubt, or at the preference of the mini-sabbatical director, a phone or video interview should be scheduled. Accepted trainees should sign a learning agreement with the mini-sabbatical director, affirming the dates, learning objectives, and expectations for both parties. The hub should provide this to the participating parties as a template document that can be customized for the specific offering. A copy of the agreement should be retained by both parties, and by the hub for record-keeping.

*Assessment and follow-up* – For the ongoing success of any mini-sabbatical hub, appropriate assessment and follow-up will be essential. At the program level, this will include periodically reviewing the catalog of offerings to determine whether any needs and/or opportunities are being missed, and whether any offerings have become obsolete and need to be removed from the catalog. Exit interviews or surveys of participating trainees will help confirm the quality of the offering. Were the learning objectives met? Was the quality of the experience commensurate with the investment of time and resources? If not, it may be necessary to retool or even abandon the particular offering. Finally, aggregation of long-term data, such as trainee publications and grants, and ongoing collaborations/mentorships between the trainee and the mini-sabbatical leader will help determine the value added by these novel experiences.

*Costs* – As a general principal, we recommend that the cost of mini-sabbaticals be borne by the trainee and/or their base institution. Such costs may be covered through institutional funds, educational dollars in the trainees’ grants, etc. Applicants for F, T, or K applications should consider including mini-sabbaticals in their training plans, and budgeting accordingly. We additionally encourage institutions to consider ways to support appropriate trainees without their own funding, who wish to participate in an “away” mini-sabbatical. In this regard, mechanisms such as the BU competitive mini-sabbatical scholarship deserve consideration. In addition, the host institution offering the mini-sabbatical should make every effort to provide the experience at little or no cost. If affiliated costs (e.g., registration fees) are necessary, these should be stated clearly and in advance. Ideally, the host institution should make a reasonable effort to provide the trainee with helpful assistance in locating an affordable place to stay – for example, providing a list of short-term housing options.

Finally, the SEQUIN group notes that, while these recommendations are intended to clarify the chain of responsibility in terms of mini-sabbatical costs, they do not adequately address the fundamental issue of lack of support for mini-sabbatical programs. We recommend that future iterations of NCATS awards and other training mechanisms consider including allocations to cover mini-sabbatical expenses.

## Toward an Integrated Network: SEQUIN, CLIC, and a National Clearinghouse for Mini-Sabbatical Offerings

Providing access to web pages at each CTSA mini-sabbatical host should prove extremely helpful for trainees seeking to find appropriate mini-sabbaticals. Nonetheless, searching through individual institutional websites for information on mini-sabbaticals will be cumbersome. A centralized system allowing trainees to find and apply to mini-sabbaticals would seem essential to the long-term success of the training endeavor, given that (1) the potential number of mini-sabbaticals and the number of host sites may be large; (2) the experiential and educational needs of trainees may be highly specific; and (3) a proper match of trainees and mini-sabbaticals will be essential to ensure appropriate access as well as sufficient utilization of offerings to ensure their ongoing availability.

With these concerns in mind, SEQUIN has paired with CLIC at the University of Rochester to create a searchable Mini-Sabbatical Opportunities Board (https://clic-ctsa.org/sequin/about). In this pilot project, functionality is limited to the identification and a description of each mini-sabbatical, along with contact information regarding how to apply. Future iterations may include links to institutional webpages, application forms, algorithmic “matching” of opportunities to candidates, and other utilities. Importantly, the CLIC system provides the capacity for hubs with mini-sabbaticals to post them directly into the listings. We encourage readers to review the current listings and to list mini-sabbaticals of their own.

## Concluding Thoughts

Through mini-sabbaticals, CTSA hubs can concisely address gaps in their own and other CTSA’s training capacities, provide opportunities for trainees to be more rounded in their skills and perspectives, and promote collaborative engagement across disciplines and institutions. But the process of creating successful mini-sabbatical programs itself requires collaboration on regional and national levels, since the mini-sabbatical offerings of any one CTSA will mainly benefit the trainees of others. For the individual mini-sabbatical, careful consideration of institutional strengths and opportunities and consistent administration will be a key to success. For the mini-sabbatical enterprise as a whole, equity between programs, common procedures for applying and participating in opportunities, easy communication across hubs, the ability of trainees to easily find the mini-sabbatical that suits them, and a minimum of red tape will prove essential for future success. As demand for mini-sabbaticals grows, cost will likely become an even greater issue, but one whose solution may eventually turn on the documentation that mini-sabbaticals effectively repay their investment in the success of trainees.

## References

[1] Institute of Medicine. The CTSA Program at NIH: Opportunities for Advancing Clinical and Translational Research. Section 4: Crosscutting Topics: Training and Education. Washington (DC): The National Academies Press; 2013 10.17226/18323. Accessed April 26, 2019. 24199260

[2] *Clinical and Translational Science Award (U54*). Retrieved from https://grants.nih.gov/grants/guide/rfa-files/RFA-TR-14-009.html Accessed August 22, 2018.

[3] *AHRQ Patient Centered Outcomes Research (PCOR) Institutional Mentored Career Development Program (K12*); 2013 Retrieved from http://grants.nih.gov/grants/guide/rfa-files/RFA-HS-13-008.html Accessed August 12, 2018.

[4] *AHRQ National Research Service Awards (NRSA) Institutional Reserach Training Grants (T32)*; 2012 Retrieved from http://grants.nih.gov/grants/guide/rfa-files/RFA-HS-12-008.html. Accessed August 12, 2018.

[5] *AHRQ Patient Centered Outcomes Research Institutional Award (K12)*. Retrieved from http://grants.nih.gov/grants/guide/rfa-files/RFA-HS-12-001.html. Accessed August 12, 2018.

[6] *AHRQ ARRA Recovery Act 2009 Limited Competition: AHRQ Institutional National Research Service Award (NRSA) Postdoctoral Comparative Effectiveness Development Training Award (T32)*; 2010 Retrieved from http://grants.nih.gov/grants/guide/rfa-files/RFA-HS-10-011.html. Accessed August 12, 2018.

[7] *AHRQ ARRA Recovery Act 2009 Limited Competition: AHRQ Mentored Clinical Scientists Comparative Effectiveness Development Award (K12)*; 2010 Retrieved from http://grants.nih.gov/grants/guide/rfa-files/RFA-HS-10-007.html. Accessed August 12, 2018.

[8] Genesis 2:3, *King James version*.

[9] Exodus 20:8–10, *King James Version*.

[10] Kimball BA. The origin of the sabbath and its legacy to the modern sabbatical. Journal of Higher Education 1978; 49(4): 303–315.

[11] Landau JL. The Sabbath. Johannesburg, South Africa: Ivri Publishing Society, 2009 2–12.

[12] Eells WCaH EV. Sabbatical leave in American higher education: Origin, early history and current practices. Bulletin (United States, Office of Education) 1962; 17: 1–75.

[13] Asenjo MA, et al Impact of sabbatical leave on hospital and university promotions. Medicina Clínica (Barc) 1998; 111(10): 378–379. 9833240

[14] Rodes J, et al Assessment of the performance of sabbatical leave: Hospital Clinic i Provincial of Barcelona (1990–1991). Research Committee of the Hospital Clinic i Provincial of Barcelona. Medicina Clínica (Barc) 1995; 104(9): 321–328. 7731299

[15] Harris IB, Wempner J. Continuing medical education reconceived: Evaluation of a sabbatical program for physicians. Academic Medicine 1996; 71(Suppl 10): S46–S48. 10.1097/00001888-199610000-00041 8940932

[16] Davidson OB, et al Sabbatical leave: Who gains and how much? Journal of Applied Psychology 2010; 95(5): 953–964. 20718526 10.1037/a0020068

[17] Soltis D. Personal and professional growth through an international sabbatical experience. American Journal of Pharmaceutical Education 2013; 77(1): 2. 23460754 10.5688/ajpe7712 PMC3578333

[18] Straus SE, Sackett DL. Clinician-trialist rounds: 26. Sabbaticals. Part 1: Should I take a sabbatical? Clinical Trials 2015; 12(2): 174–176. 25480539 10.1177/1740774514562917

[19] Mulder DS. Thoracoscopy in Europe: Views from North America. The Annals of Thoracic Surgery 1993; 56(3): 731–733. 8379781 10.1016/0003-4975(93)90964-j

[20] Gibb BC. The two-week sabbatical. Nature Chemistry 2011; 3(7): 495–496. 10.1038/nchem.1080 21697862

[21] Boyatzis RE. Transforming Qualitative Information: Thematic Analysis and Code Development. Thousand Oaks, CA: SAGE Publishing, 1998.

[22] *Mini Sabbatical Award 2018*; 2018 Retrieved from https://www.bu.edu/ctsi/2017/12/04/mini-sabbatical-award-2018-2/. Accessed August 12, 2018.

